# In-depth comparison of methods to isolate RNA from common human cell lines

**DOI:** 10.1186/s12864-025-12497-7

**Published:** 2026-01-13

**Authors:** Thomas Josef Zech, Robert Fürst

**Affiliations:** Pharmaceutical Biology, Deparmtent of Pharmacy – Center for Drug Research, Ludwig-Maximilians-Universität München, Butenandtstraße 5-13, Munich, 81377 Bavaria Germany

**Keywords:** RNA isolation, RNA yield, RNA integrity, RNA purity, gDNA contamination

## Abstract

RNA isolation is a fundamental procedure that is universally applied in biomedical research. The isolation of high-quality and high-purity RNA is essential for many biochemical analyses, including RT-qPCR and RNA sequencing. However, this isolation can be challenging due to the instability of RNA, its structural similarity to DNA, and the complexity of biological samples. Numerous methods and kits have been developed and marketed to surmount these challenges. To this day, most studies comparing these methods for RNA isolation have focused on samples that are especially difficult to process, such as FFPE tissue. In contrast, few studies have compared different RNA isolation procedures using simpler samples, such as mammalian cell cultures. Moreover, most studies employ outdated and/or incomplete analysis techniques or only compare a minimal set of methods. This article presents a thorough comparison of various manual RNA isolation methods, with a focus on spin column-based kits. The isolated RNAs were evaluated regarding yield, integrity, and purity using state-of-the-art techniques: photometric analysis, automated electrophoresis-based integrity measurement, and qPCR. While most kits performed satisfactorily, our results highlight some outliers, particularly regarding genomic DNA contamination and RNA integrity. This comprehensive analysis provides researchers with valuable data to support selecting an appropriate RNA isolation method.

**Supplementary Information:**

The online version contains supplementary material available at 10.1186/s12864-025-12497-7.

## Introduction

Ribonucleic acid (RNA) molecules serve diverse and crucial functions in biology, particularly in gene expression. Accordingly, measuring and analyzing the quantity and properties of RNA is indispensable to biological research. To do this, RNA is usually isolated before most analysis methods are performed. For successful RNA isolation, many factors are critical, among them RNA integrity, yield, and purity. RNA integrity, for example, is crucial for methods that rely on intact RNA, e.g. direct RNA-seq [1]. This is challenging due to the low stability of RNA [2] and ubiquitous RNases, which degrade the RNA if not properly avoided or inactivated [3]. Purity is crucial, because contaminants can interfere with downstream processes. For example, a common contaminant is genomic deoxyribonucleic acid (gDNA). Many RNA analysis methods require the reverse transcription (RT) of RNA into cDNA. However, gDNA can behave similarly to cDNA, which can reduce measurement accuracy, e.g. in sequencing applications [4]. Moreover, additional common contaminants like guanidinium thiocyanate can be PCR inhibitors [5]. Another key metric is the yield: Getting more RNA out of limited material allows for more input into the respective analysis method, which leads to a more sensitive detection, especially of low-abundance transcripts. Multiple RNA isolation methods have been developed to address these challenges. The technique developed by Chirgwin et al. [6] was one of the first to allow high-quality isolation of RNA. However, laborious CsCl gradient ultracentrifugation was required to purify the RNA after lysis. A significant improvement was introduced by Chomczynski and Sacchi [7, 8], who developed a "single step" method based on lysis in phenol/guanidinium thiocyanate and subsequent organic phase extraction. This method is highly effective and much more time-efficient, with variants still in use today. Nonetheless, the reliance on toxic and difficult-to-handle chemicals, especially phenol and chloroform, provides significant safety concerns for users. In response, spin-column-based RNA isolation methods based on RNA’s property to bind to silica membranes were developed [9]. These kits are available from various companies and are commonly used for many applications. Moreover, silica-covered magnetic beads, which work similarly to the spin columns, have also been marketed. Concurrently with the development of RNA isolation methods, RNA analysis methods have also undergone significant improvements. For example, the ratio of ribosomal RNA bands to each other on an agarose gel was initially used as an indicator of high RNA quality. Today, RNA integrity analysis is based on the entire electropherogram, e.g., by calculating an RNA integrity number equivalent (RINe), which offers a much more accurate estimation of RNA quality [10]. Therefore, multiple studies have been performed to confirm whether currently available kits and/or methods can isolate high-quality RNA. However, most of these studies focus on the isolation of RNA from complex samples, for example, wastewater [11], tissue [12–14], bacteria [15, 16], plants [17], and more. Interestingly, few studies compare the quality of isolated RNA from mammalian cell culture, e.g. [18, 19]. Moreover, some studies only compare a limited number of methods, such as Sultan et al. [18], who compared two different methods with each other. Additionally, outdated techniques for assessing the quality of the isolated RNA are still used. For example, in the publication by Ortega-Pinazo et al. [19] RNA-integrity was assessed using standard agarose gel electrophoresis, and gDNA contamination was not quantified at all. Accordingly, we aimed to fill this data gap and, to the best of our knowledge, performed the most extensive comparison of RNA isolation methods from cultured mammalian cells currently available, utilizing state-of-the-art analysis techniques. Interestingly, we found some unexpected differences between various methods, particularly in terms of RNA integrity and the presence of contaminating gDNA.

## Material and methods

### Cell culture

All cells were grown in saturated humidity at 37 °C and 5 % CO$_2$. Human umbilical vein endothelial cells (HUVEC) were purchased from PromoCell (Heidelberg, Germany) and grown in endothelial cell growth medium (ECGM) enhanced (PELOBiotech, Martinsried, Germany) supplemented with all included supplements (except gentamicin/amphotericin B) plus 100 U/ml penicillin, 100 $\upmu$g/ml streptomycin, 2.5 $\upmu$g/ml amphotericin B (all PAN-Biotech, Aidenbach, Germany) and 10 % FCS (Anprotec, Bruckberg, Germany). For HUVEC cultivation, cell culture plates and flasks were coated with collagen G (Sigma-Aldrich, St. Louis, MO, USA) before the cells were seeded onto them. THP-1 cells were obtained from the DSMZ (German Collection of Microorganisms and Cell Cultures, Leipzig, Germany) and cultured in RPMI-1640 (Anprotec) containing 10 % FCS (Anprotec). HeLa and HEK-293T cells were also obtained from the DSMZ and cultivated in DMEM (Anprotec) containing 10 % FCS (Anprotec). For HeLa cells, 5 % non-essential amino acids and 2 mM glutamine (both Pan-Biotech) were added to the medium. Cells were generally split every 2-4 days at a ratio of 1:3. For detachment of adherent cells (HUVEC, HEK-293T, and HeLa), the cells were carefully washed twice using PBS and then incubated with 1x trypsin/EDTA (T/E, Pan-Biotech) for 30 s to 5 min, until all cells were detached. For experimental purposes, 200 000 HeLa, 200 000 HEK293T, 300 000 HUVECs, or 250 000 THP-1 were seeded onto each well of a 6-well plate and incubated for three days before lysis and RNA isolation. Cell counts were determined using a Vi-CELL XR automated cell counter (Beckmann-Coulter, Brea, CA, USA).

### RNA isolation

In general, cells were lysed on day three after seeding, and the lysates were stored at -80 °C for one day before RNA isolation, to simulate a lab scenario in which immediate processing might be impossible. Adherent cells were washed twice using PBS+ and then lysed directly in the well, whereas THP-1 cells were spun down, washed, and then lysed in a microcentrifuge tube. Then, the RNA was isolated mostly according to the manufacturers’ instructions; if steps were optional but recommended, they were performed unless otherwise specified. Any deviation from the manufacturers’ instructions will be mentioned in the following. As a general point, we have also tried to minimize the use of toxic substances. This means that if the manufacturers’ instructions specifically recommended adding $\beta$-mercaptoethanol ($\beta$-ME) to the lysis buffer, it was done. Still, when the use of dithiotreitol (DTT) or other reducing agents was allowed, we opted for DTT. None were added if the manufacturers’ instructions did not list $\beta$-ME, DTT, or other reducing agents as recommended additives for the lysis buffer. The additives to the lysis buffers for the respective kits are listed in Table 1. For DNase digestion, the DNase included with or recommended for each kit was used, if applicable. However, no specific DNase set was available and/or recommended for some kits. These kits were the GeneJet RNA purification Kit (Thermo Fisher, Waltham, MA, USA), the FastGene RNA Basic Kit (Nippon Genetics Europe, Düren, Germany), and the Simply Total RNA Isolation Kit (GeneDireX, Inc., Taichung City, Taiwan). We used the RNase-free DNase Set (Qiagen, Hilden, Germany) analogously to the RNeasy Mini kit for these kits. For this, the first washing step with the second washing buffer was typically split into two, with a 15-minute on-column DNase digestion step in between. A more precise description can be found in the respective section of each kit. We typically used 50 $\upmu$l of the included elution buffer or RNase-free water to elute the purified RNA, as this was the recommended volume in most protocols. The only exception was the FastGene RNA Premium Kit, for which the recommended elution volume was 20 $\upmu$l, although a range of 10–50 $\upmu$l can be used according to the manual. The elution volumes and buffers can be found in Table 2. To visualize all column-based kits, images were recorded using a smartphone camera (Samsung Electronics, Suwon, Korea).

**Table 1:** Additives and their concentration in the lysis buffer used for each isolation method described in this study

Isolation kit	Additives to the lysis buffer
PureLink RNA Mini-Kit	$\beta$-ME 1%
MagMAX *mir*Vana total RNA isolation kit	isopropanol 50%,$\beta$-ME 0,5%
GeneJET RNA purification Kit	DTT 40 mM
RNeasy Mini Kit	DTT 40 mM
Monarch Total RNA Miniprep Kit	none
FastGene RNA Basic Kit	DTT 40 mM
FastGene RNA Premium Kit	DTT 40 mM
Simply Total RNA Isolation Kit	none
Quick-RNA MiniPrep	none
innuPREP RNA Mini Kit 2.0	none
TRI-Reagent	none

**Table 2:** Additives and their concentration in the lysis buffer used for each isolation method described in this study

Isolation kit	Elution buffer	Elution volume
PureLink RNA Mini-Kit	RNase-free water	50$\upmu$l
MagMAX *mir*Vana total RNA isolation kit	Elution buffer (pre-heated)	50$\upmu$l
GeneJET RNA purification Kit	RNase-free water	50$\upmu$l
RNeasy Mini Kit	RNase-free water	50$\upmu$l
Monarch Total RNA Miniprep Kit	RNase-free water	50$\upmu$l
FastGene RNA Basic Kit	Buffer RE (elution buffer)	50$\upmu$l
FastGene RNA Premium Kit	Buffer RE (elution buffer)	20$\upmu$l
Simply Total RNA Isolation Kit	REB (elution buffer)	50$\upmu$l
Quick-RNA MiniPrep	RNase-free water	50$\upmu$l
innuPREP RNA Mini Kit 2.0	RNase-free water	50$\upmu$l
TRI-Reagent	RNase-free water	50$\upmu$l

#### PureLink RNA Mini-Kit

For isolation of RNA without DNase digestion, the PureLink RNA Mini-Kit (Thermo Fisher) was used according to the manufacturer’s instructions, skipping all DNase digestion steps. For RNA isolation with DNase digestion, the manufacturer’s instructions were followed for both types of DNase digestion. For on-column DNase digestion, the PureLink DNase set (catalog number 12185010, Thermo Fisher) was used according to the provided instructions. For off-column DNase digestion, DNase I amplification grade (catalog number 18068015, Thermo Fisher) was used according to the instructions provided with the isolation kit. The sample for off-column digestion was taken from the eluted RNA obtained from the procedure without prior DNase digestion. After off-column digestion, the DNase was removed by additional column purification on PureLink RNA mini columns.

#### MagMAX *mir*Vana total RNA isolation kit

For the isolation of RNA with the MagMAX *mir*Vana kit (Thermo Fisher), the manufacturer’s protocol was followed for the manual isolation. For isolation without DNase digestion, the DNase digestion step was skipped; for isolation with DNase digestion, it was included.

#### GeneJET RNA purification Kit

For isolation of RNA using the GeneJET RNA purification Kit (Thermo Fisher) without DNase digestion, the manufacturer’s instructions for isolation of RNA from mammalian cultured cells were followed. For isolation with DNase digestion, after step 5 of the manual (700 $\upmu$l Wash Buffer 1), the columns were washed with 350 $\upmu$l Wash Buffer 2. Then, 10 $\upmu$l DNase (Qiagen) and 70 $\upmu$l Buffer RDD (Qiagen) were added to the columns and incubated for 15 min at RT. The columns were then rewashed with 350 $\upmu$l Wash Buffer II. We then continued with Step 7 of the manual (a wash with 250 $\upmu$l Wash Buffer II). For elution, the protocol of the GeneJET RNA purification kit recommends two elution steps with 50 $\upmu$l. However, preliminary experiments in our lab showed that the amount of RNA obtained in the second elution step was usually negligible. Therefore, only one elution step was performed.

#### RNeasy Mini Kit

For isolation with the RNeasy Mini Kit (Qiagen), we followed the manufacturer’s protocol for cultured mammalian cells using spin technology. When DNase digestion was performed, it was performed using the RNase-Free DNase Kit (Qiagen) according to the instructions of the RNeasy Mini Kit.

#### Monarch Total RNA Miniprep Kit

The Monarch Total RNA Miniprep Kit (New England Biolabs, NEB, Ipswich, MA, USA) was used according to the manufacturer’s instructions. When DNase digestion was performed, it was performed using the included DNase according to the instructions. When it wasn’t performed, this step was skipped.

#### FastGene RNA Basic Kit

For isolation without DNase digestion, the FastGene RNA Basic Kit (Nippon Genetics Europe) was used according to the manufacturer’s instructions, following the standard protocol. When DNase digestion was performed, step 6 was split into two washes with buffer RW2 at 350 $\upmu$l each, with a 15-minute on-column incubation in between the two washing steps with 10 $\upmu$l DNase solution and 70 $\upmu$l buffer RDD (Qiagen).

#### FastGene RNA Premium Kit

As the FastGene RNA Premium Kit’s (Nippon Genetics Europe) protocol always includes DNase digestion, we omitted step 10 and onward for the isolation without DNase digestion. In contrast, the complete protocol was followed when DNase digestion was performed.

#### Simply Total RNA Isolation Kit

The Simply Total RNA isolation Kit (GeneDireX, Inc., Taichung City, Taiwan) was obtained from Genaxxon Bioscience (Ulm, Germany). The protocol of the Simply Total RNA isolation kit recommends a DNase digestion procedure. However, the necessary DNase was not available to us. Therefore, we performed the DNase digestion with the RNase-Free DNase set (Qiagen). To do this, instead of washing the column with 600 $\upmu$l W2 buffer, the columns were washed with 350 $\upmu$l W2 buffer. Then, 10 $\upmu$l DNase solution and 70 $\upmu$l buffer RDD (Qiagen) were added to the column and incubated for 15 min at RT. Afterward, the columns were rewashed using 350 $\upmu$l of buffer 2. Then, the manufacturers’ protocol was followed again.

#### Quick-RNA MiniPrep

For isolation of RNA using the Quick-RNA MiniPrep Kit (ZymoResearch, Irvine, CA, USA), the manufacturer’s protocol was followed. For samples including DNase digestion, the DNase digestion step in the protocol was performed using the DNase supplied with the kit; for samples without DNase digestion, the DNase digestion step was skipped.

#### innuPREP RNA Mini Kit 2.0

For RNA purification using the innuPREP RNA Mini Kit 2.0 (iST Innuscreen GmbH, Ebnat-Kappel, Switzerland) without DNase digestion, the manufacturers’ instructions for RNA extraction from eukaryotic cells were followed. For additional DNase digestion, the DNase set available separately from Innuscreen was applied using the on-column protocol provided with the DNase set.

#### TRI-Reagent

As a comparison to the spin kits, isolation of RNA using TRI-Reagent (Thermo Fisher) was also performed. To do this, the manufacturers’ instructions were followed. Isolation using TRI-reagent or similar methods typically results in very low gDNA contamination, even without DNase treatment. Therefore, and because we used this isolation method mainly as a point of reference for the largely column-based kits, no DNase digestion was performed.

### Analysis of RNA yield, protein contamination, and organic compound contamination

To quantify the RNA yield, the protein contamination, and the organic compound contamination, we used a NanoDrop One device (Thermo Fisher). First, the elution buffer or water used for elution, whichever was provided in the respective kit (Table 2), was measured as a blank. Then, each RNA sample was measured. The absorbance value at 260 nm was used to calculate the concentration in ng/$\upmu$l, which was done automatically by the device. This value was multiplied by the elution volume to calculate the total yield per isolation. The ratio of absorbance at 260 nm/230 nm was used to estimate contamination with organic compounds such as buffer components, and the ratio of absorbance at 260 nm/280 nm was used to estimate contamination with proteins and aromatic compounds. The results of the concentration measurements and absorbance ratios were also collected in the supplementary table.

### Analysis of RNA quality

To assess the quality of the isolated RNA, the RNA integrity number equivalent (RINe) was measured using the RNA ScreenTape (Agilent Technologies, Santa Clara, CA, USA) on a TapeStation 4150 (Agilent Technologies). To do this, the RNA samples were diluted to approximately 250 ng/$\upmu$l and then processed per the manufacturer’s instructions. Briefly, 1 $\upmu$l diluted sample was mixed with 5 $\upmu$l sample buffer, heated to 72 °C, cooled down, and then transferred into the TapeStation to be analyzed. The RNA ScreenTape ladder (Agilent Technologies) was used as a size reference. The results of the Tapestation analysis were also collected in the supplementary table.

### Analysis of gDNA contamination

To analyze the contamination with gDNA, a genomic RNase P quantifying qPCR assay (TaqMan copy number reference assay, RNase P, Catalog number 4403326, Thermo Fisher) was used on a QuantStudio 3 device (Thermo Fisher) with the Luna Universal Probe qPCR Master Mix (NEB). The cycling conditions were 1 min incubation at 95 °C followed by 45 cycles of 15 s denaturing at 95 °C and 30 s annealing, extension, and fluorescence reading at 60 °C. As we wanted to measure absolute gDNA contamination, a concentration curve of human CEPH DNA (Thermo Fisher) was measured simultaneously. Measurements were performed in triplicate. The total gDNA contamination was then normalized to the total RNA yield. As the gDNA contamination varied considerably between the different kits, the top 3 kits for each cell type (with and without DNase treatment) were also depicted separately to improve readability. The results of the qPCR measurements were also collected in the supplementary table.

### Processing duration

For all kits, the time needed to isolate RNA manually from 18 samples was estimated once (including DNase digestion, except for TRI-Reagent) using a mobile phone watch (Samsung Electronics) and documentation of start and stop times. Processing times were rounded to the nearest 5-minute mark.

### Data processing and graphs

R (version 4.4.2), including the tidyverse package collection (version 2.0.0), was used for data processing and the generation of the graphs. $N=3$ for all methods of RNA isolation, with the only exceptions being the RNeasy Mini Kit and the GeneJET RNA purification Kit when RNA was isolated from HUVECs and DNase digestion was performed. In that case, $N=6$ and $N = 5$ respectively. No statistical analysis was performed.

## Results

### RNA yield

**Fig. 1:**
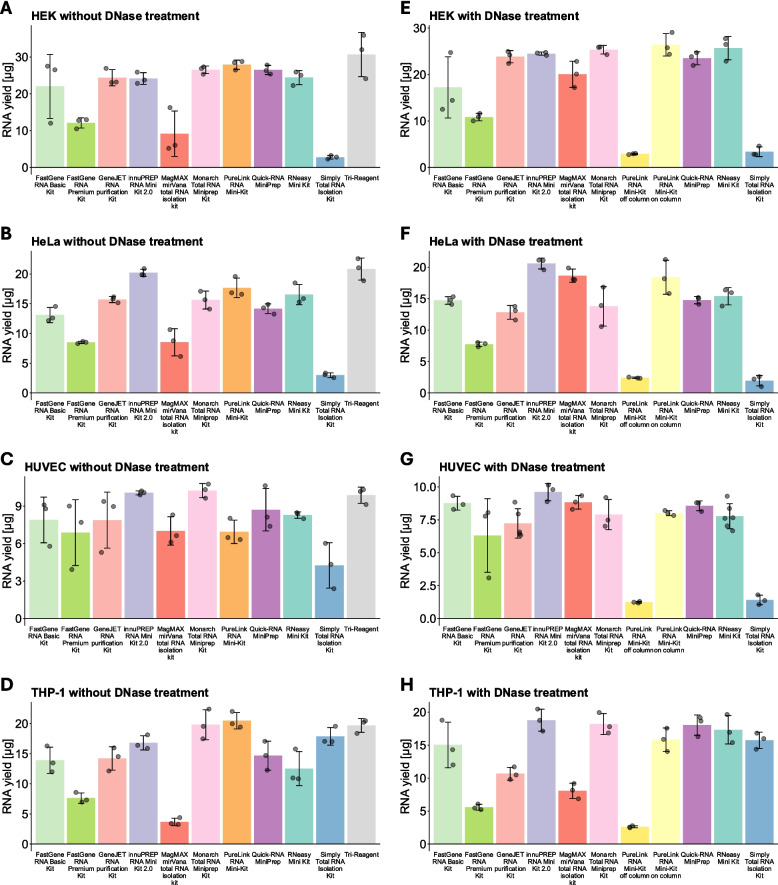
RNA yields of different isolation Methods. Depiction of RNA yields achieved using the labeled isolation kits without a DNase digestion step (**A**–**D**) or with a DNase digestion step (**E**–**H**) in the cell types HEK293T (**A**, **E**), HeLa (**B**, **F**), HUVEC (**C**, **G**), and THP-1 (**D**, **H**). Data are depicted as mean ± SD

The RNA yield varied depending on the cell type and applied RNA isolation method, as can be seen in Fig. 1. Overall, apparent yields for some kits were slightly higher in samples without DNase treatment (Fig. 1A–D in comparison to E–H). There were only minor differences between the kits when RNA was isolated without DNase digestion, except for the MagMAX *mir*Vana total RNA isolation kit, the FastGene RNA Premium Kit, and the Simply Total RNA Isolation Kit. These kits isolated lower amounts of total RNA, especially from the cell lines, while the results in HUVECs did not differ as much. The Simply Total RNA Isolation Kit consistently achieved low yields, except in THP-1 cells, where the yields were comparable to the other well-performing kits (Fig. 1D, H). The results of RNA isolated with DNase digestion were largely consistent with the results without DNase digestion, with a few notable exceptions: The PureLink RNA Mini-Kit achieved a lower yield when off-column DNase digestion was performed compared to on-column digestion or no DNase digestion (Fig. 1E–H vs A–D). Moreover, the MagMAX *mir*Vana total RNA isolation kit achieved higher yields when DNase digestion was performed compared to when DNase digestion wasn’t performed (Fig. 1E–H vs A–D).

### RNA purity (proteins/organic compounds)

**Fig. 2:**
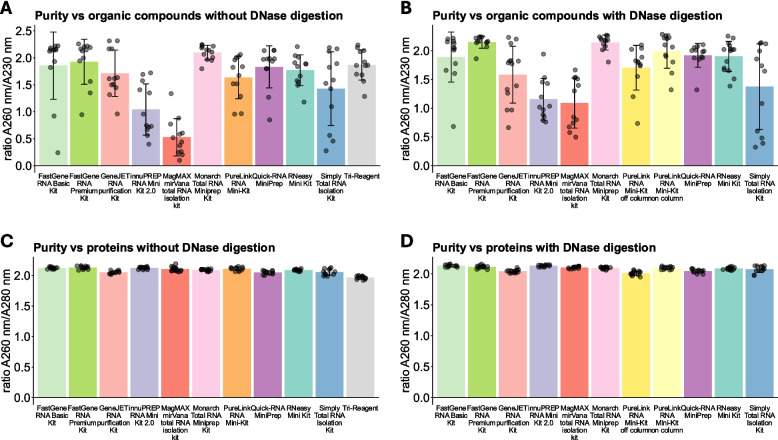
Absorbance measurements indicative of organic compound and protein contamination. Spectra of the isolated RNAs were recorded on a nanophotometer. **A**, **B** The ratio of absorbance at 260 nm/230 nm was calculated to measure purity vs. non-aromatic organic compounds. **C**, **D** The ratio of absorbance at 260 nm/280 nm was calculated to measure protein and aromatic compound contamination. Data are depicted as mean ± SD

Typically, two main kinds of contaminants can be found in isolated RNA: genomic DNA (gDNA) and proteins originating from the sample material, and carryover of buffer components from the isolation procedure [20]. Protein and organic compound contamination can be measured through UV absorbance. Because RNA possesses comparatively low absorbance at 230 nm and 280 nm, which is usually around 2 times lower than at 260 nm for completely pure RNA [21], the ratio of the absorbance at 260 nm/230 nm can be used to estimate organic compound contamination and 260 nm/280 nm can be used to estimate the amount of protein or aromatic compound contamination. As can be seen in Fig. 2, the amount of non-aromatic contaminants, likely stemming from carryover of buffers, varied between kits (Fig. 2A, B). Some variance is expected, especially when performing isolation manually. However, this might be mitigated through optimized column design and well-established protocols. We considere the Monarch Total RNA Miniprep Kit to be a positive example, because it provided consistent results: The A260 nm/A230 nm results were always above 1.8 (Fig. 2A, B). This was also the case for the FastGene RNA Premium Kit, but only when DNase digestion was performed (Fig. 2B). On the other side of things, the MagMAX *mir*Vana kit, the innuPREP RNA Mini Kit 2.0, and the Simply Total RNA Isolation Kit had low and/or inconsistent A260 nm/A230 nm ratios, indicating high amounts of buffer carryover (Fig. 2A, B). In contrast, none of the kits seemed to have issues with protein or aromatic compound contamination (Fig. 2C, D).

### RNA purity (gDNA)

**Fig. 3:**
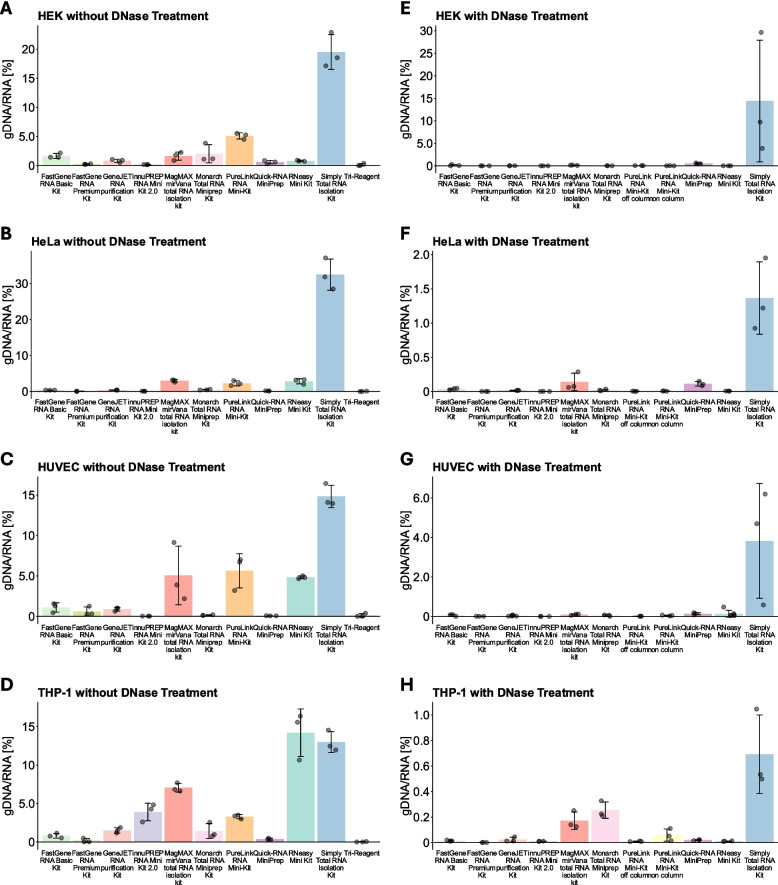
Measurement of relative gDNA contamination. gDNA contamination was measured using qPCR and then divided by the RNA yield of each sample. **A**–**D** shows data from cell lines of the listed kits when no DNase digestion was performed, **E**–**H** shows data from cell lines of the listed kits when DNase digestion was performed. Data are depicted as mean ± SD

**Fig. 4:**
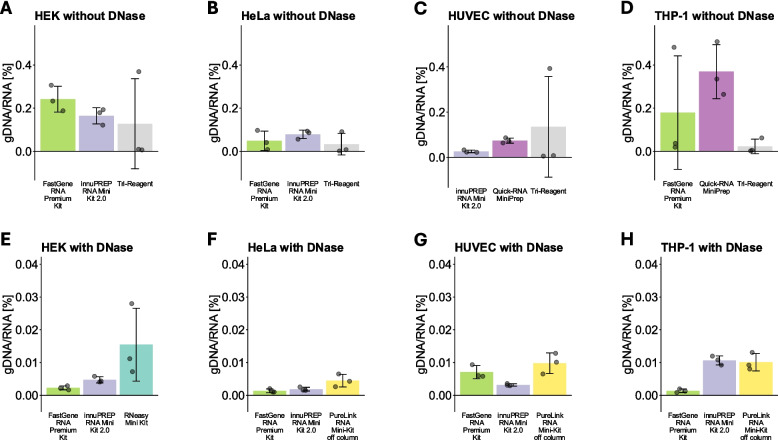
Separate graphs for the kits with the lowest amount of gDNA contamination. The best three kits from Fig. 3 were graphed separately. **A**–**D** show the best three kits in the labeled cell lines when no DNase digestion was performed, **E**–**H** show the best three kits when DNase digestion was performed. Data are depicted as mean ± SD

Some seemingly large differences were observed in the relative gDNA contamination, as can be seen in Fig. 3. What is most noticeable in Fig. 3A–D is the Simply Total RNA Isolation Kit, which provided high amounts of gDNA contamination: up to 30 % of the measured RNA was gDNA when no DNase digestion was performed. Because the high amounts of gDNA contamination made it difficult to visually evaluate the differences in the kits with lower amounts of contamination, we also depicted the results of the three kits with the lowest amounts of gDNA contamination per cell type separately in Fig. 4. Moreover, we additionally provide all numerical values in the supplementary table. We observed that TRI-Reagent-based RNA isolation was consistently among the three isolation methods with the lowest gDNA contamination when no DNase digestion was performed. The other two spots were interchangeably occupied by the FastGene RNA Premium Kit, the innuPREP RNA Mini Kit 2.0, and the Quick-RNA Mini Kit (Fig. 4A–D). However, the overall gDNA contamination could be reduced even more when DNase digestion was performed (Fig. 3E–H). The Simply Total RNA Isolation Kit still provided comparatively high gDNA contamination compared to the other kits, between 0.5 and 10 %, depending on the cell type (Fig. 3E–H). However, it must be stated that the DNase digestion was not performed as recommended in the protocol, because the proprietary DNase set was not available to us. Therefore, these results should be interpreted with caution. In accordance with this, the best performing kits all had a corresponding DNase set (Fig. 4E–H). The innuPREP RNA Mini Kit 2.0 and the FastGene RNA Premium Kit were consistently among the three kits with the lowest amounts of gDNA contamination for all cell types tested, while the other spot was either occupied by the RNeasy Mini Kit or the PureLink RNA Mini-Kit when the DNase was applied off-column. Interestingly, when the DNase digestion was performed on-column, the PureLink RNA Mini-Kit did not perform as well as when the digestion was performed off-column (Figs. 3E–H, 4E–H).

### RNA integrity

**Fig. 5:**
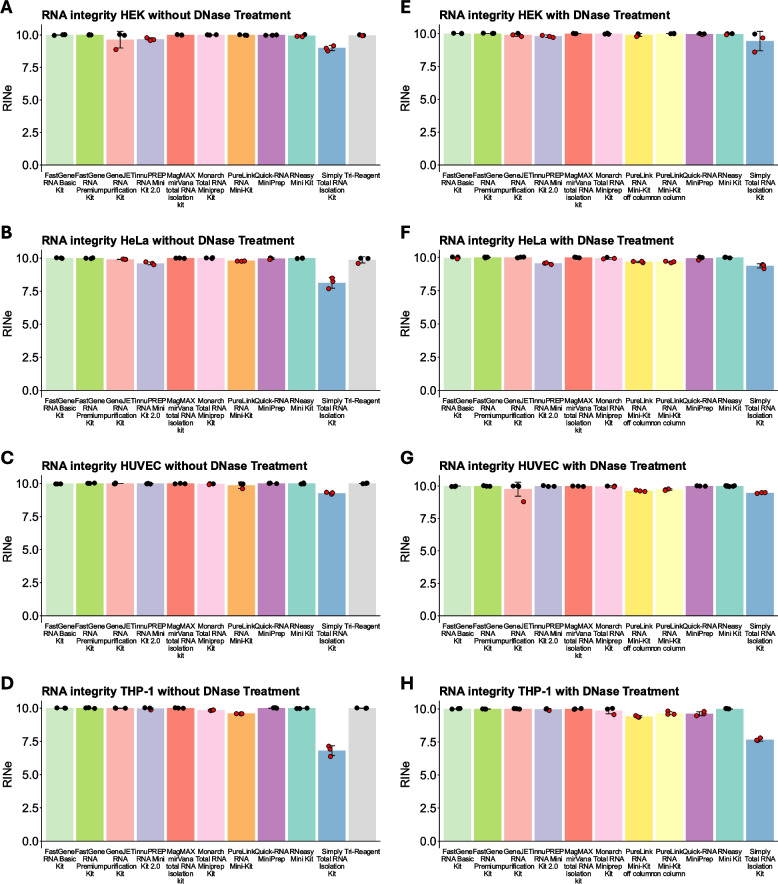
RNA integrity measurements. **A**–**H** RINe values of the indicated samples of RNA isolated from the indicated cell lines with the indicated kits without (**A**–**D**) or with (**E**–**H**) DNase digestion. All individual values $< 10$ are marked red. Data are depicted as mean ± SD

Arguably, the most important property of isolated RNA is its integrity, which we estimated through measurement of the RNA integrity number equivalent (RINe) [10]. A RINe of 10 indicates perfectly intact RNA, and a RINe of 1 indicates totally degraded RNA. As we isolated RNA from cell culture, we did not expect to isolate low-integrity RNA. Therefore, we wanted to visualize how consistently kits facilitate the isolation of highest quality RNA with a RINe $= 10$. Accordingly, all dots with a RINe $< 10$ are marked in red (Fig. 5). Only the FastGene RNA Premium Kit and the MagMAX *mir*Vana total RNA isolation kit achieved a RINe of 10 in every single sample (Fig. 5). Most other kits performed generally well, but a few measurements revealed a RINe $< 10$. For most, this was only a few isolated samples; however, the innuPREP RNA Mini Kit 2.0, the PureLink RNA Mini-Kit, and the Monarch Total RNA Miniprep Kit all had at least one condition in which the RINe was consistently $< 10$. The Simply Total RNA Isolation Kit seemed to be unable to isolate high-integrity RNA, especially in THP-1 cells, where the RINe was consistently $< 7.5$ (Fig. 5D, H).

### Processing duration

The time it took to manually isolate RNA from 18 samples (including DNase digestion, except for TRI-Reagent) is listed in Table 3. Generally, kits with an on-column DNase digestion were quicker to process than kits with an off-column DNase digestion, because the separate elution and re-isolation steps are not necessary. TRI-Reagent requires longer centrifugation times, which is why this process also takes comparatively long. Additionally, the MagMAX *mir*Vana total RNA isolation kit takes a longer time, as the collection of the beads on the magnets is slow, although this may vary depending on the magnet used, and because pipetting must be done more carefully.

**Table 3:** Manual processing times

Isolation Kit	Processing time [h:mm]
PureLink RNA Mini-Kit (on-column)	2:05
PureLink RNA Mini-Kit (off-column)	2:40
MagMAX *mir*Vana total RNA isolation kit	3:00
GeneJET RNA purification Kit	1:40
RNeasy Mini Kit	1:40
Monarch Total RNA Miniprep Kit	1:30
FastGene RNA Basic Kit	1:20
FastGene RNA Premium Kit	2:00
Simply Total RNA Isolation Kit	2:30
Quick-RNA MiniPrep	1:30
innuPREP RNA Mini Kit 2.0	2:15
TRI-Reagent	2:00

## Discussion

### RNA yield

In general, most kits isolated similar amounts of RNA overall. Importantly, we did not correct for the cell count between the samples immediately before lysis, which might have introduced some variation that cannot be accounted for in hindsight. Therefore, some differences in yields might be due to differences in cell proliferation or similar factors, rather than being a property of the respective kit. The Simply Total RNA Isolation Kit consistently yielded lower amounts in adherent cells. In contrast, the yields appeared comparable to most other kits in the suspension cell-line THP-1 (Fig. 1D, H). Even though a substantial part of this higher yield was gDNA (Fig. 3D, H), this still indicates that the lower yield in the other cell types was most likely not a consequence of a lower binding capacity of the columns. Instead, we hypothesize that it is probably a result of insufficient lysis of adherent cells. The protocol recommends collecting any type of cell in a microcentrifuge tube before lysis and does not indicate that lysis in the well is possible. Therefore, the low yields achieved with this kit are likely due to the change we made to the protocol. However, since direct lysis of adherent cells in the well is much faster and more convenient, we still consider this a disadvantage. Other kits that achieved comparatively low yields were the FastGene RNA Premium Kit (Fig. 1), the MagMAX *mir*Vana total RNA isolation kit, when no DNase digestion was performed (Fig. 1A–D), and the PureLink RNA Mini-Kit when off-column DNase digestion was performed (Fig. 1E–H). For the FastGene RNA Premium Kit, we think some of the RNA might be lost due to the elution and re-isolation steps required for the off-column DNase digestion. Additionally, the lower elution volume compared to other methods may also have resulted in less efficient elution of the RNA. While the quick guide of the kit recommends 20 $\upmu$l, which is why we used this elution volume, the longer form manual only specifies that 10–50 $\upmu$l can be used. Accordingly, increasing the elution volume might improve the yields achieved with this kit. For the MagMAX *mir*Vana total RNA isolation kit and the PureLink RNA Mini-Kit, the respective low yields are most likely a consequence of how we performed the isolation. The protocol of the MagMAX *mir*Vana total RNA isolation kit does provide an option for skipping the DNase digestion step, which we did nonetheless. Since the samples for which we skipped DNase digestion are the only samples in which this kit achieved low yields, we believe this is the main reason and not indicative of any problem with the kit. For the PureLink RNA Mini-Kit, the off-column DNase digestion protocol allows 80 $\upmu$g of RNA as an input, however, only in a volume of 8 $\upmu$l. Since we used 8 $\upmu$l of RNA from the samples processed without DNase digestion, the concentrations and total amount of RNA we could use were limited. Therefore, higher yields are likely possible when the RNA is eluted in a lower volume before DNase digestion. In contrast, no kit achieved extraordinarily high yields. However, because a limited amount of cells was used in this study, we likely provided much less RNA than the binding capacity for most kits.

### RNA contamination

#### Absorbance measurements

Absorbance measurements are usually used to estimate yield and purity for every individual sample of isolated RNA. Typically, low A260 nm/A230 nm ratios are a consequence of buffer carryover. In contrast, a low A260 nm/A280 nm ratio can indicate the presence of contaminating proteins or aromatic chemicals, such as phenol [21], which can absorb UV light strongly at both 230 nm and 280 nm. A low A260 nm/A230 nm value, and accordingly buffer carryover, is often a consequence of user errors. However, there are certain qualities a kit can possess that may reduce the probability of these errors. For example, protocol optimizations such as an additional dry centrifugation step before elution can be helpful for spin-column-based isolation kits. Moreover, an optimized column insert design may reduce the buffer retained on or atop the purification column. In our study, there were four main kinds of column insert designs for RNA purification columns: Type 1 was a silica column held in place from the top by a rubber or plastic ring and from the bottom by a funnel-like design of the plastic insert with plastic bridge-like structures partially crossing the silica column multiple times. This design appears to allow for a mostly free flow of the buffer out of the column; however, we sometimes observed buffer retention on the rubber ring. Type 2 was very similar, but without the plastic bridges below the silica column. Instead, a porous material covered the entire surface of the bottom of the silica column. Type 3 was also very similar, but instead of bridges or a porous substance, a perforated plastic plate held the column in place from the bottom. For type 4, the silica column is held in place from the bottom through a funnel-like design with small bridges covering a tiny part of the silica column. From the top, there appears to be no additional fixation. Table 4 classifies all applied spin-column kits into one of the described types. Moreover, a collection of images of all columns can be found in the supplementary images. Overall, we achieved mostly good absorbance ratios at 260 nm/230 nm when using spin column-based kits and TRI-Reagent (Fig. 2A, B). The main exception was the innuPREP RNA Mini Kit 2.0, which only achieved a value of around 1 on average (Fig. 2A, B). As described in Table 4, the silica column is fixed in a type 3 design. However, in contrast to other type 3 designs, there are fewer perforations (five total), and the plastic covers a comparatively large portion of the silica column (refer also to the supplementary images). This might lead to increased buffer retention and explain the observed results. As an example for a well-performing kit, the Monarch Total RNA Miniprep Kit achieved A260 nm/A230 nm ratios of around 2.0 for every single sample (Fig. 2A, B). We again believe that the column design, type 4 in this case (Table 4), might explain these results: Only four very small plastic bridges hold the silica column in place from the bottom. Otherwise, the only other thing obstructing the flow of the buffer appears to be the silica membrane itself. Therefore, buffer retention and carryover are likely minimal, which we believe could lead to the consistently low buffer carryover we observed. Another very well-performing kit was the FastGene RNA Premium Kit, but only when DNase digestion was performed. For this kit, we believe that the second isolation step after the first elution might facilitate the removal of previously retained buffers by using a completely fresh column, which would also explain why the results were less reproducible when DNase digestion wasn’t performed. When isolating RNA with the MagMAX *mir*Vana total RNA isolation kit, which is based on magnetic beads, we had issues fully removing the wash buffers without disturbing the beads, which reflected in the lower A260 nm/A230 nm ratios (Fig. 2A, B). However, because the MagMAX *mir*Vana total RNA isolation kit is optimized for automatic processing instruments, such as the KingFisher (Thermo Fisher) series, we believe that better A260 nm/A230 nm ratios are likely achievable when performing fully automated extractions. Nonetheless, in our setup, we could not achieve A260 nm/A230 nm ratios comparable to most centrifugation-based RNA isolation methods. In contrast to the A260 nm/A230 nm ratios, none of the methods described had issues with the A260 nm/A280 nm ratio (Fig. 2C, D). This indicates that all methods efficiently removed proteins from the sample. Moreover, phenol carryover, which might be expected from methods such as the TRI-Reagent, also appeared to be no problem.

**Table 4:** Types of column designs of the spin-column-based isolation kits

Kit name	Column design type
PureLink RNA Mini-Kit	Type 2
GeneJET RNA purification Kit	Type 1
RNeasy Mini Kit	Type 2
Monarch Total RNA Miniprep Kit	Type 4
FastGene RNA Basic Kit	Type 3
FastGene RNA Premium Kit	Type 1 (RNA binding column), Type 3 (RNA mini-elute column)
Simply Total RNA Isolation Kit	Type 1
Quick-RNA MiniPrep	Type 2
innuPREP RNA Mini Kit 2.0	Type 3

#### gDNA

Since DNA is structurally highly similar to RNA, isolated RNA often contains residual gDNA. However, in contrast to photometric analysis of RNA, measurements of residual gDNA contamination have to be performed separately, for example, through qPCR without reverse transcription (-RT-qPCR) [22]. Moreover, most downstream methods used to analyze RNA are based on cDNA and thus might also measure contaminating gDNA, potentially skewing the results. For this reason, an important part of the MIQE guidelines is that qPCR without reverse transcription (-RT-qPCR) should be performed for each sample and assay, to confirm that no relevant gDNA contamination is present [23]. Additionally, gDNA, or other kinds of contaminating DNA like cell-free DNA, can also alter the results of RNA-seq if not thoroughly removed [24]. This is because DNA can also be amplified and modified by common RNA-seq library preparation protocols [25]. In the sequencing experiment, this can lead to confounding results, e.g. decreased detection of exonic sequences and increased detection of intronic and intergenic sequences [26] and increase the false discovery rate [25]. Therefore, we believe that applying RNA isolation methods that consistently isolate RNA with low amounts of gDNA contamination could help mitigate this issue. Accordingly, we measured the total amount of gDNA contamination by performing -RT-qPCR. The most noticeable result we observed was the high amounts of gDNA contamination when using the Simply Total RNA Isolation Kit, which was $>10 \%$ on average for all cell types when no DNase digestion was performed (Fig. 3A–D). This could sometimes be reduced when DNase digestion was performed; however, the amount of gDNA contamination still appeared to be higher than in all other methods (Fig. 3E–H). Besides this, the PureLink RNA Mini-Kit, the RNeasy Mini Kit, and the MagMAX *mir*Vana total RNA isolation kit also had considerable amounts of gDNA contamination when no DNase digestion was performed (Fig. 3A–D). In contrast, the Quick-RNA Mini Kit, the FastGene RNA premium Kit, the innuPREP RNA Mini Kit 2.0, and TRI-Reagent all provided comparatively low amounts of gDNA contamination of $< 0.5 \%$ without DNase digestion (Fig. 3A–D). These kits employ techniques to separate RNA and DNA based on their physical and/or chemical properties. The Quick-RNA Mini Kit, the FastGene RNA Premium Kit, and the innuPREP RNA Mini Kit 2.0 use a gDNA removal column, which preferentially binds gDNA, before the RNA is isolated. The higher density of DNA for the TRI-Reagent causes it to be located in the interphase, while RNA is located in the top phase. This suggests that methods to reduce DNA without DNase may be at least somewhat effective. Accordingly, if RNA isolation without DNase digestion is preferred, using a gDNA removal column or similar processes is strongly recommended. However, our data revealed that additional DNase digestion can further reduce the amount of residual gDNA, as most kits provided low amounts of gDNA contamination when DNase digestion was performed. The main exception, the Simply Total RNA Isolation Kit, still provided comparatively high amounts of gDNA. Additionally, the MagMAX *mir*Vana total RNA isolation kit and the Quick-RNA Mini Kit had comparatively high amounts of gDNA contamination in HeLa and THP-1 cells (Fig. 3D, H), whereas the Monarch Total RNA Isolation Kit and the PureLink RNA Mini-Kit (on-column) provided comparatively high amounts of gDNA contamination in THP-1 cells (Fig. 3H) when DNase digestion was performed. In contrast, four kits consistently provided very low amounts of gDNA contamination: The FastGene RNA Premium kit and the innuPREP RNA Mini Kit 2.0 were among the three kits with the lowest average amount of gDNA contamination in all cell types, typically achieving $\le$ 0.01 % residual gDNA contamination (Fig. 4E–H). In addition to these two kits, the RNeasy Mini Kit (Fig. 4E) and the PureLink RNA Mini-Kit, when off-column DNase digestion was performed (Fig. 4F–H), were able to provide very low levels of residual gDNA contamination. Multiple manufacturers state that off-column DNase digestion results in lower gDNA contamination than other methods of gDNA removal, likely because DNA bound to a column is less accessible for the DNase. Our data seems to partially confirm this, because only two of the spin-column kits were used with this approach, and both provided some of the lowest amounts of gDNA contamination. Importantly, while we only tested the PureLink RNA Mini-Kit and the FastGene RNA Premium Kit using an off-column DNase digestion protocol, many of the other kits, such as the RNeasy Mini-Kit or the Simply Total RNA Isolation Kit, also offer equivalent protocols. Therefore, similarly low amounts of gDNA contamination could potentially be achieved with most kits. However, out of all the spin column-based kits tested in this study, the FastGene RNA Premium Kit is the only one that already includes the material for removing the DNase after the off-column digestion. For others, an additional column often has to be used for each sample if column purification is desired after DNase digestion. Alternatively, the DNase can be heat-inactivated; however, this might lead to unreliable A260 nm/A280 nm measurements. Moreover, it also introduces an additional point of potential user error, as insufficient heating results in residual DNase activity, while overheating decreases RNA integrity [27]. The MagMAX *mir*Vana total RNA isolation kit, as a non-spin-column-based kit, also utilizes DNase digestion in solution and includes the required cleanup material. Despite this, the residual DNA levels were comparatively high, at least in HeLa and THP-1 cells (Fig. 3F, H). While we do not have a sufficient explanation for this, we believe the issue we had with buffer retention when using this kit might play a role. Buffer components such as guanidinium thiocyanate might inhibit DNase activity through their chaotropic properties, and therefore could increase gDNA contamination. Accordingly, we believe this kit might perform better when RNA isolation is performed automatically. Our data show that thorough gDNA removal is also possible when DNase digestion is performed on-column. Especially the innuPREP RNA Mini-Kit 2.0 appears to be able to remove DNA very efficiently, indicating that off-column DNase digestion is not strictly superior to on-column protocols. This kit utilizes a combination of a gDNA removal column and on-column DNase digestion, which may contribute to the efficient gDNA removal. However, the Monarch Total RNA Miniprep Kit and the Quick-RNA Miniprep Kit also feature this combination, but appeared to remove DNA less efficiently. Moreover, the RNeasy Mini Kit only utilizes DNase digestion on-column, without a gDNA removal column, and still performed well in this metric. Accordingly, we consider a well-optimized DNase digestion method to be more important than a gDNA removal column. We also show that DNase digestion protocols are at least sometimes compatible between different kits, because the RNase-free DNase Set was also able to efficiently remove DNA contamination in the FastGene RNA Basic Kit and the GeneJET RNA purification Kit (Fig. 3), without obvious impacts on other RNA quality metrics.

### RNA integrity

Another critical property of RNA is its integrity, which is crucial for complex analysis methods such as long-read RNA-seq. Usually, RNA integrity is very high when isolating RNA from mammalian cell culture. While cells, for example immune cells such as THP-1 [28], express RNases, these are usually inactivated efficiently by chaotropic and reducing agents in lysis buffers [29, 30]. Therefore, we expected most kits to perform well overall, and thus primarily intended to evaluate how consistently perfect RNA integrity was achieved, as indicated by a RINe $= 10$. We were surprised to find that the Simply Total RNA Isolation Kit provided consistently low RINe values, which were even $<7.5$ in THP-1 cells. While the DNase digestion step, which was not the correct one for this kit, might have influenced this value, the Simply Total RNA Isolation Kit did not perform better when DNase digestion wasn’t performed. Other than that, the PureLink RNA Mini-Kit and the innuPREP RNA Mini Kit 2.0 both provided a RINe $< 10$ in multiple cell types. Additionally, the Monarch Total RNA Miniprep Kit provided a RINe $< 10$ in all three samples of THP-1 cells when no DNase digestion was performed, and the Quick-RNA MiniPrep kit provided a RINe $< 10$ in all three samples of THP-1 cells when DNase digestion was performed. Most other kits only had a few isolated samples, in which the RINe was not perfect. The two main exceptions were the MagMAX *mir*Vana total RNA isolation kit and the FastGene RNA Premium Kit, which achieved a RINe $= 10$ in every single sample. Crucially, RNA degradation can originate not only from residual RNases of the original cell but also from improper handling. Therefore, even when high-quality kits are used, we recommend working in a separate room with clean, RNase-free working surfaces and pipetting tips.

## Conclusion

Our study suggests that there are differences in the quality of RNA, even when isolated from seemingly simple samples, such as cultured mammalian cells. Overall, this shows that RNA quality should not be taken for granted, even when using commercially available kits and isolating from relatively simple materials. To facilitate choosing an appropriate kit, we created a summary table (Table 5). Regardless of the kit or method researchers choose, we recommend routine checks for RNA integrity and purity, especially when performing high-effort techniques like RNA-seq.

**Table 5:** Summary of the properties of each individual kit

Kit name	Advantages	Disadvantages
PureLink RNA Mini-Kit (on-column)	Low DNA contamination when using off-column DNase	$\beta$-ME in the lysis buffer, DNase not included
MagMAX *mir*Vana total RNA isolation kit	automation friendly, very consistent RNA integrity	Difficult manual processing, requires $\beta$-ME in the lysis buffer
GeneJET RNA purification Kit	Works well with RNase-free DNase Set	No specific DNase set available
RNeasy Mini Kit	Quick processing, high purity, low gDNA contamination when used with Qiagen DNase set	DNase not included
Monarch Total RNA Miniprep Kit	Very consistent elimination of organic contaminants, comparatively low gDNA contamination without DNase step	comparatively high gDNA contamination with DNase digestion for THP-1
FastGene RNA Basic Kit	Fast processing, worked with RNase-free DNase set	No DNase set specifically available
FastGene RNA Premium Kit	Consistent integrity, low gDNA and buffer contamination	Lower yields, longer processing times than most kits
Simply Total RNA Isolation Kit	simple processing	low integrity, high amounts of gDNA contamination, low yields in adherent cells
Quick-RNA MiniPrep	comparatively low gDNA contamination without DNase digestion	comparatively high amounts of gDNA contamination for HeLa cells
innuPREP RNA Mini Kit 2.0	low gDNA contamination	less consistent integrity and purity over buffer components than competitors
TRI-Reagent	very low gDNA contamination without DNase digestion	handling of toxic chemicals required

## Supplementary Information

Supplementary Material 1.

Supplementary Table.

## Data Availability

All raw data used for the creation of the figures can be found in the supplementary table.

## Abbreviations

CEPH: Centre d’Etude du Polymorphisme Humain
DNA: Deoxyribonucleic acid
ECGM: Endothelial cell growth medium
FCS: Fetal calf serum
FFPE: Formalin fixed and paraffin embedded
gDNA: Genomic DNA
HUVEC: Human umbilical vein endothelial cell
NEB: New England Biolabs
PBS: Phosphate buffered saline
PCR: Polymerase chain reaction
RINe: RNA-integrity number equivalent
RNA: Ribonucleic acid
RNA-seq: Next-generation RNA sequencing
RT: Reverse transcription
qPCR: Quantitative PCR
SD: Standard deviation
T/E: Trypsin and EDTA
