# Supplementary material for: In-depth comparison of methods to isolate RNA from common human cell lines
Source: BMC Genomics. 2026 Jan 13;27:100. doi: 10.1186/s12864-025-12497-7 (PMC12836834; doi:10.1186/s12864-025-12497-7)
Supplement: Supplementary file 1 — Supplementary Material 1. [file 12864_2025_12497_MOESM1_ESM.zip › supplementary images.pdf]

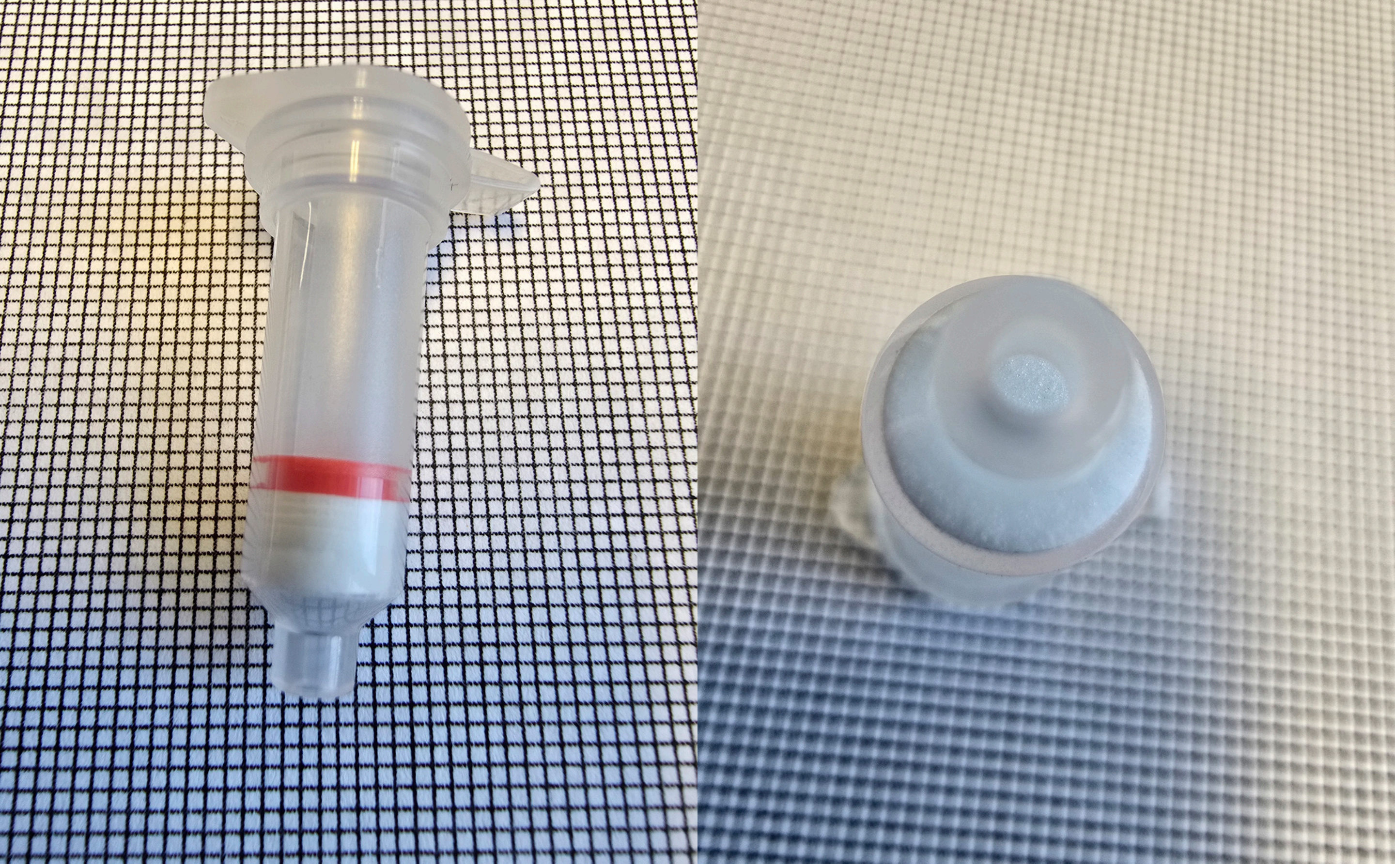

PureLink RNA Mini-Kit column
From left to right: Spin Cartridge, bottom of the Spin Cartridge

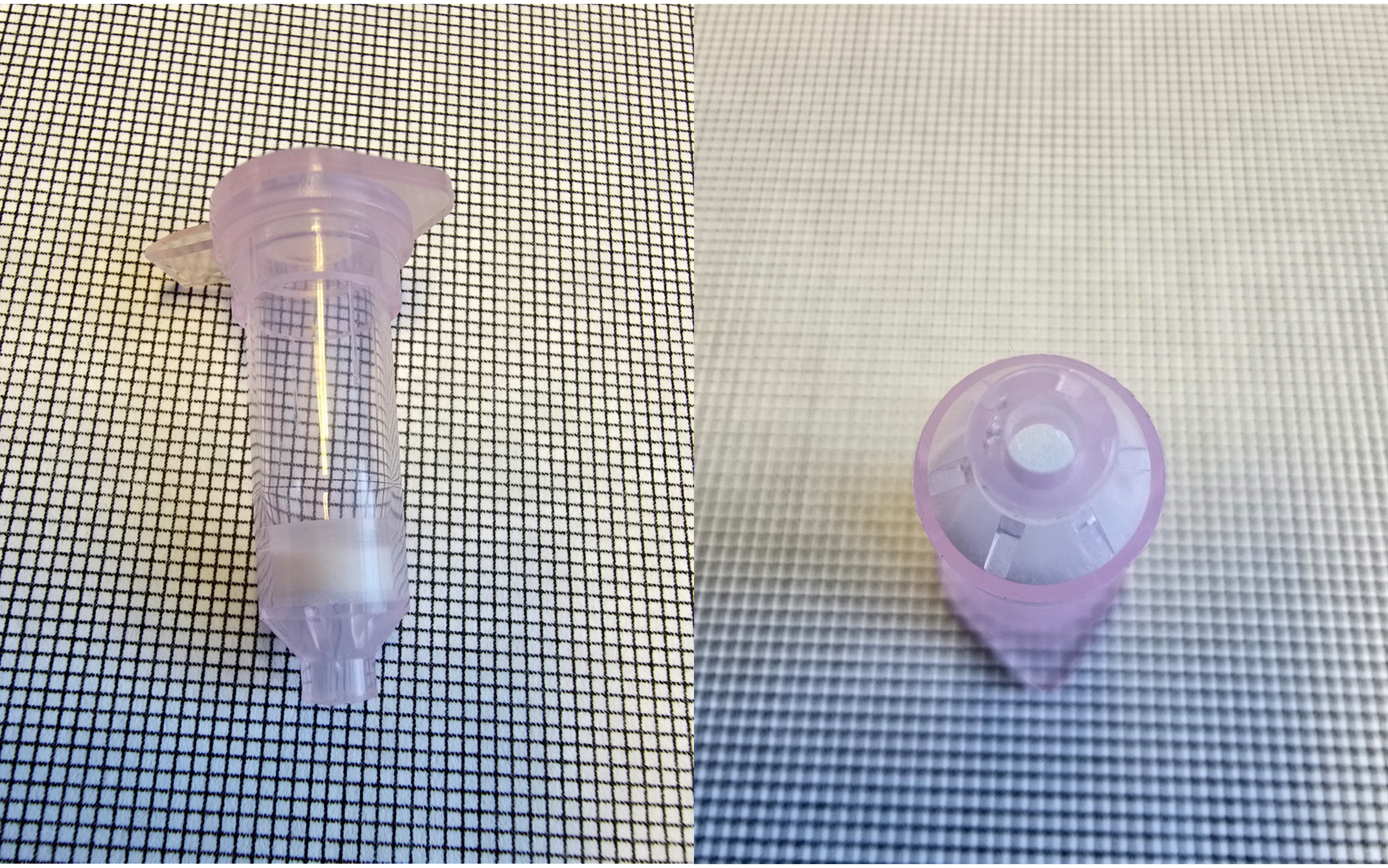

GeneJET RNA purification Kit column
From left to right: GeneJET RNA Purification Column, bottom of the GeneJET RNA Purification Column

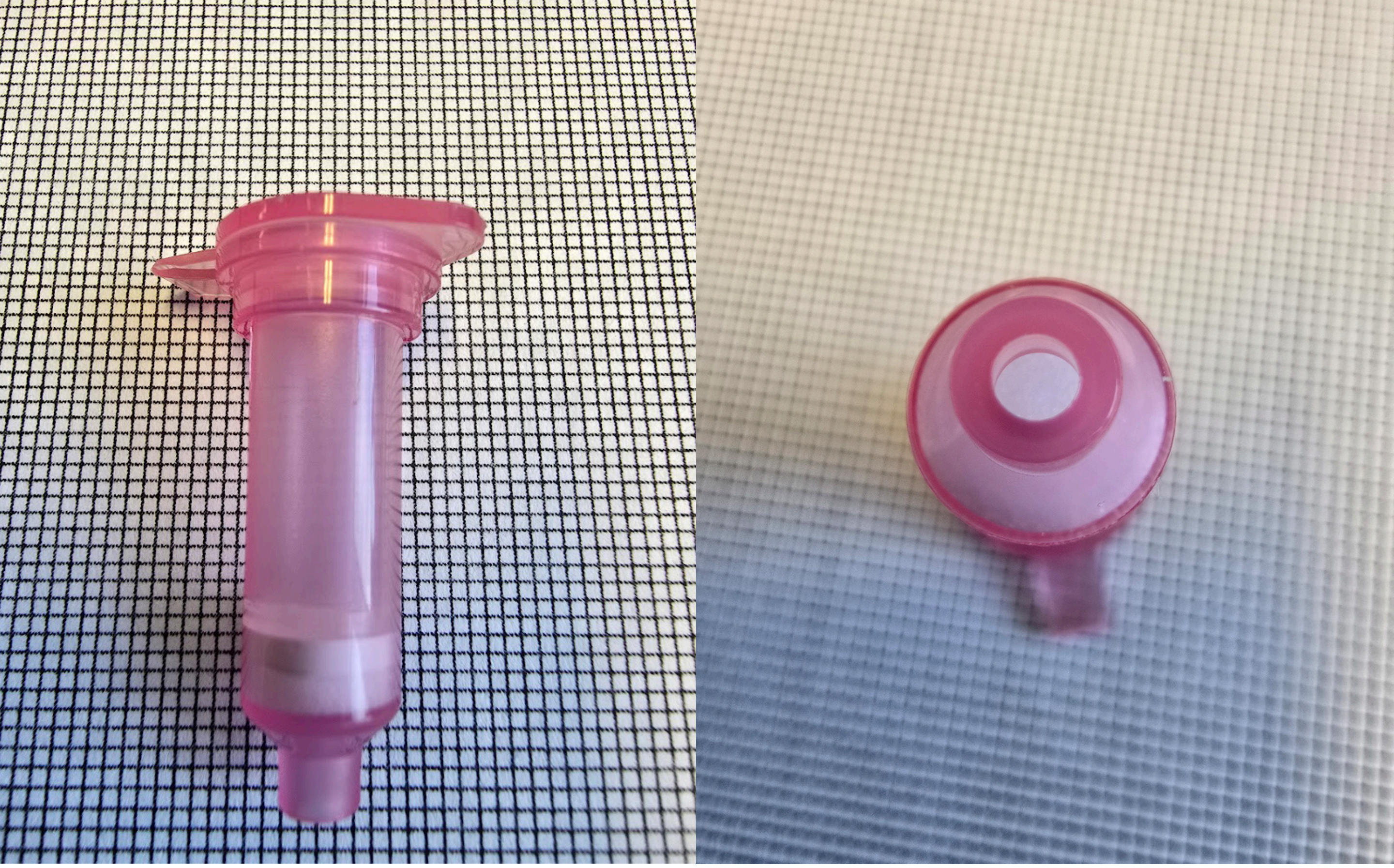

RNeasy Mini Kit column
From left to right: Rneasy Mini Spin Column, bottom of the RNeasy Mini Spin Column

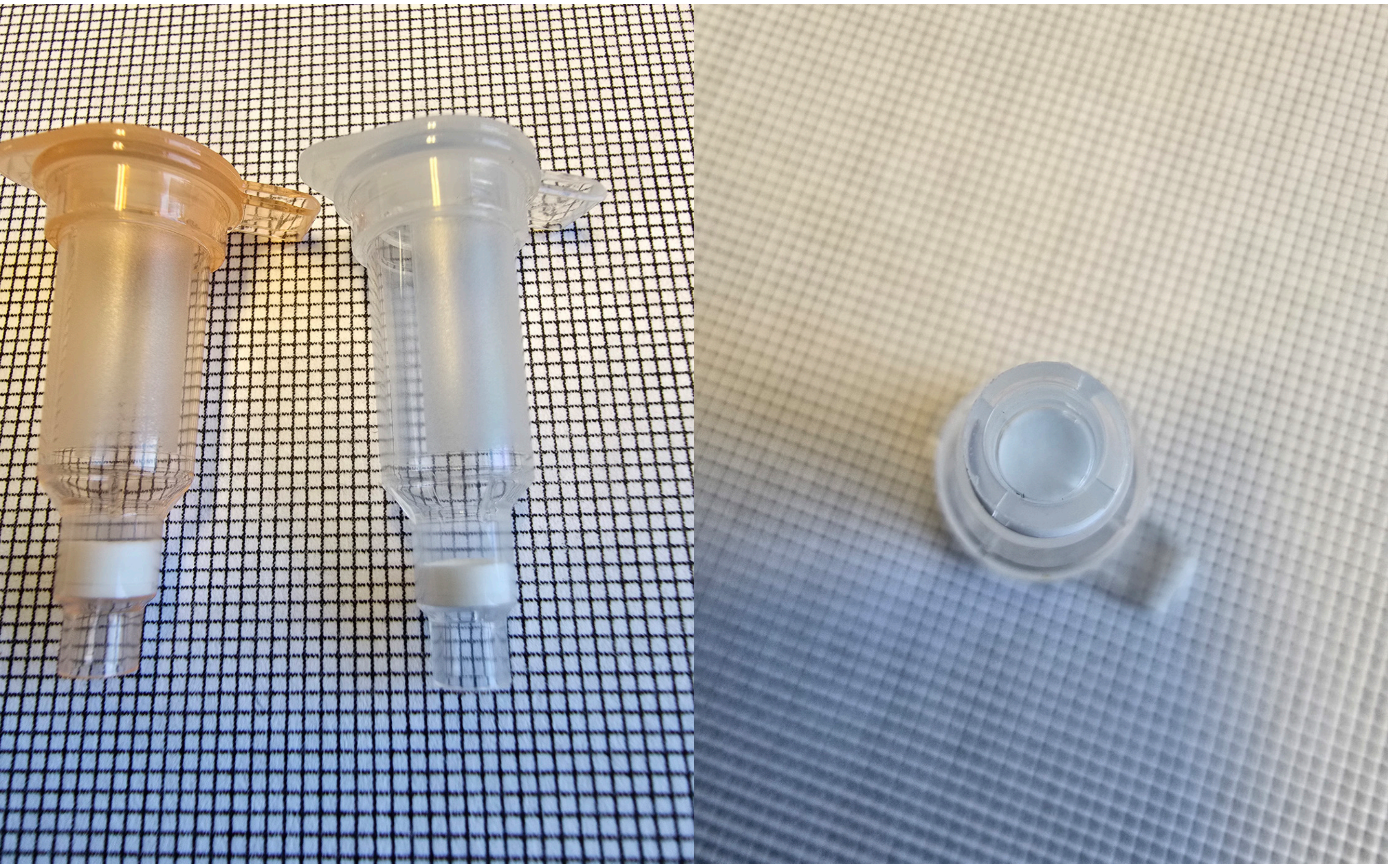

Monarch Total RNA Miniprep Kit columns
From left to right: Monarch gDNA Removal Column, Monarch RNA Purification Column, bottom of the Monarch RNA Purification Column

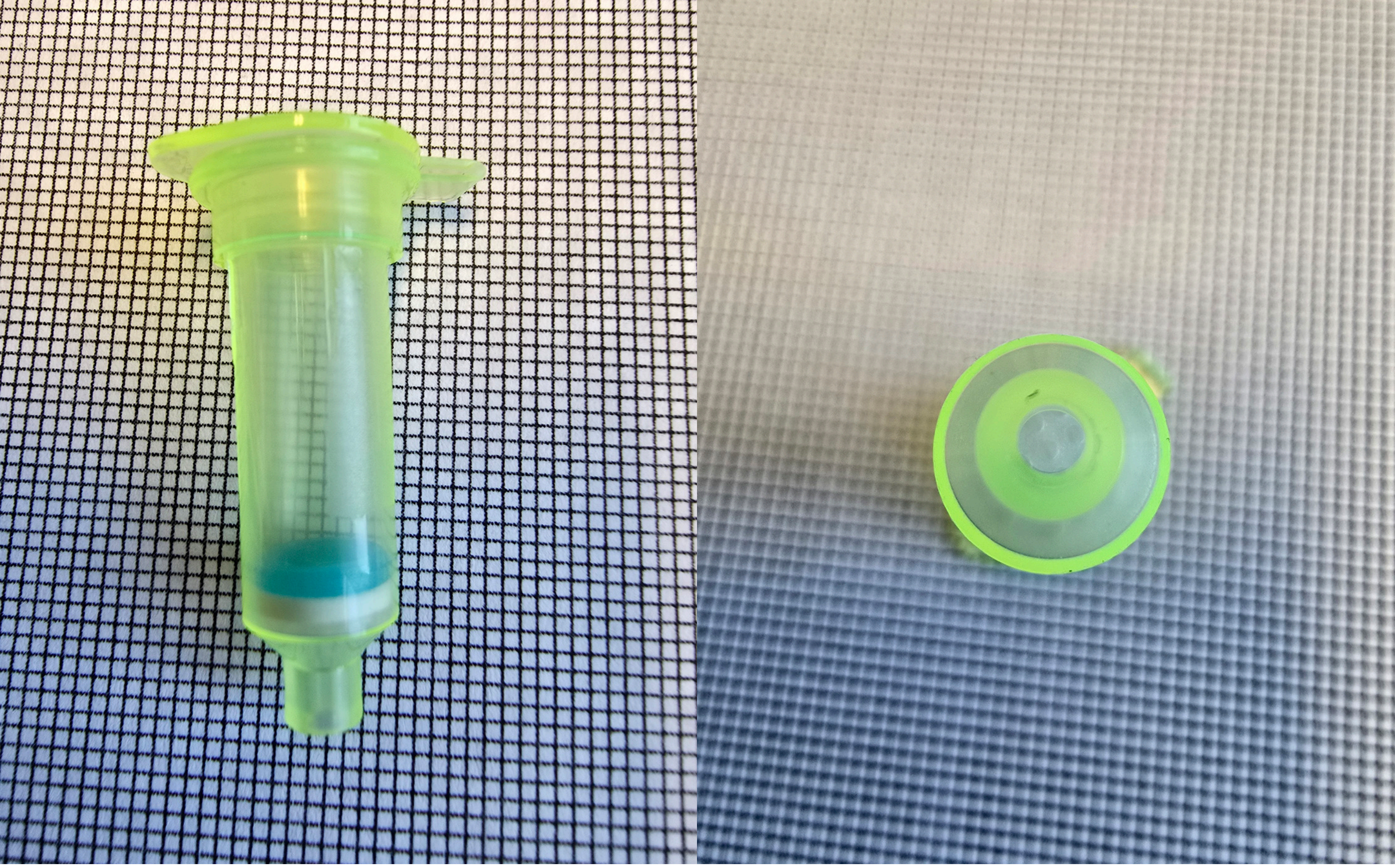

FastGene RNA Basic Kit column
From left to right: FastGene RNA binding column, FastGene RNA binding column, bottom of the FastGene RNA binding column

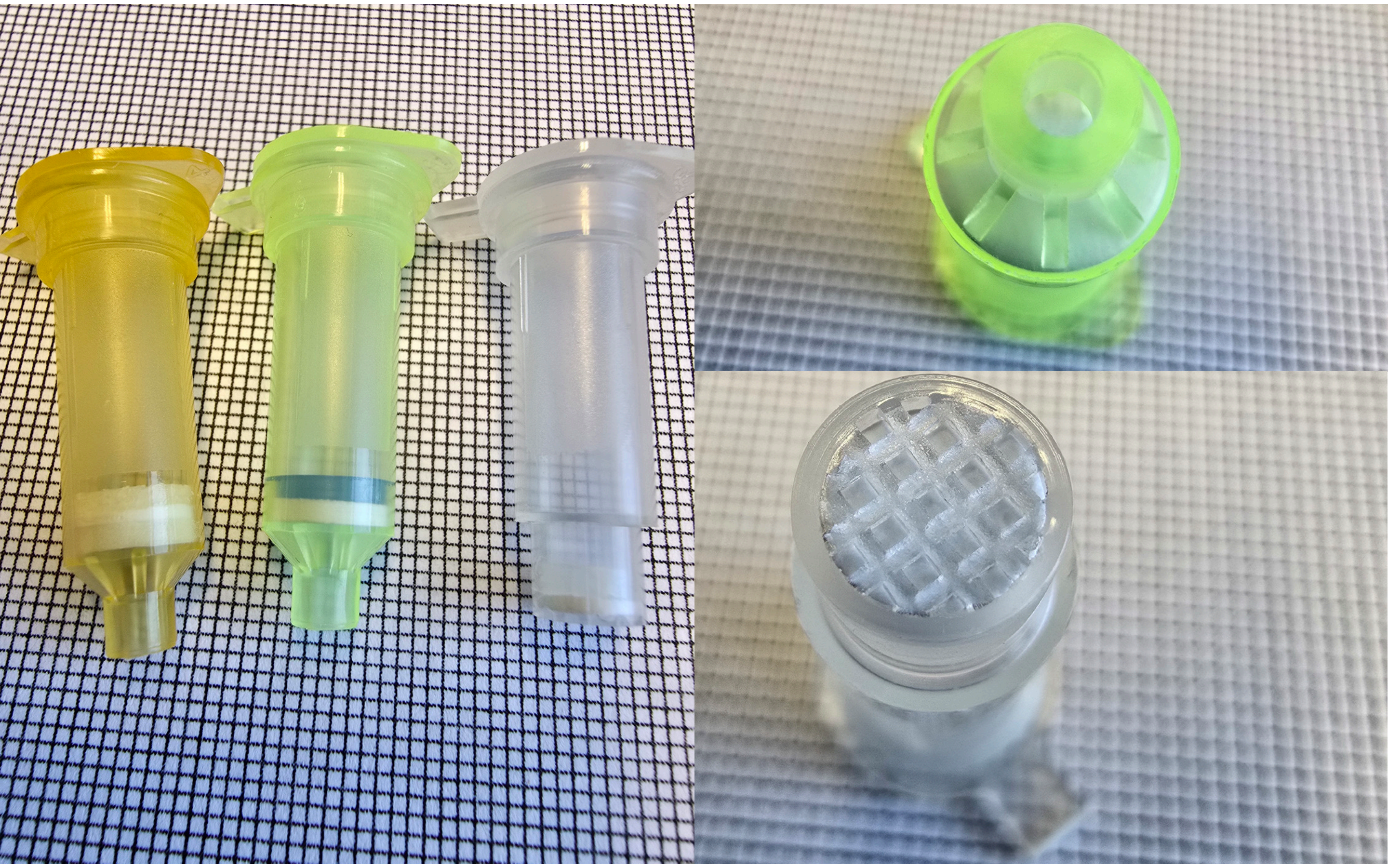

FastGene RNA Premium Kit columns
From left to right: FastGene RNA filter column, FastGene RNA binding column, FastGene RNA mini-elute column, bottom of the FastGene RNA binding column (top right), bottom of the FastGene RNA mini-elute column (bottom right)

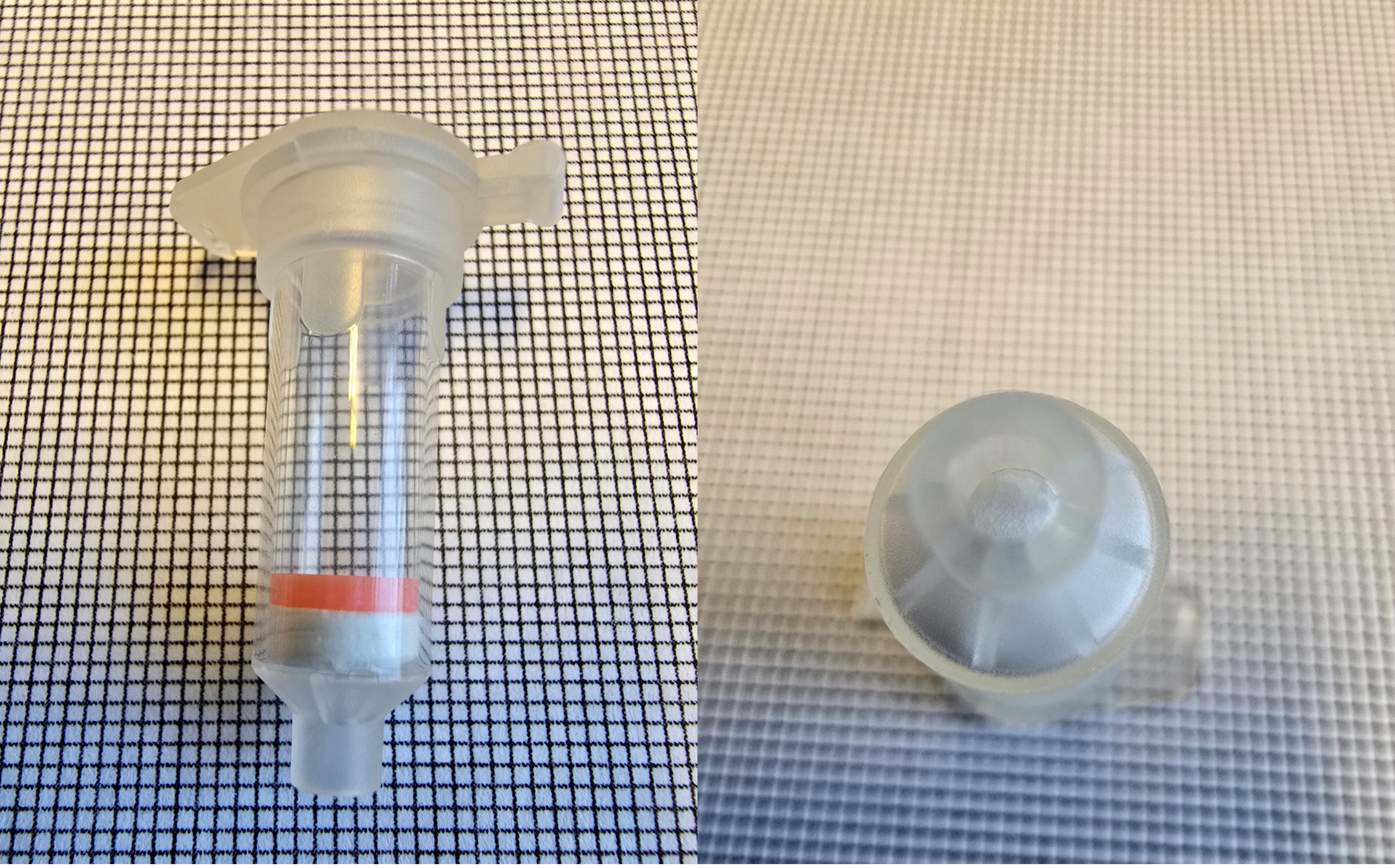

Simply Total RNA Isolation Kit column
From left to right: DR column, bottom of the DR column

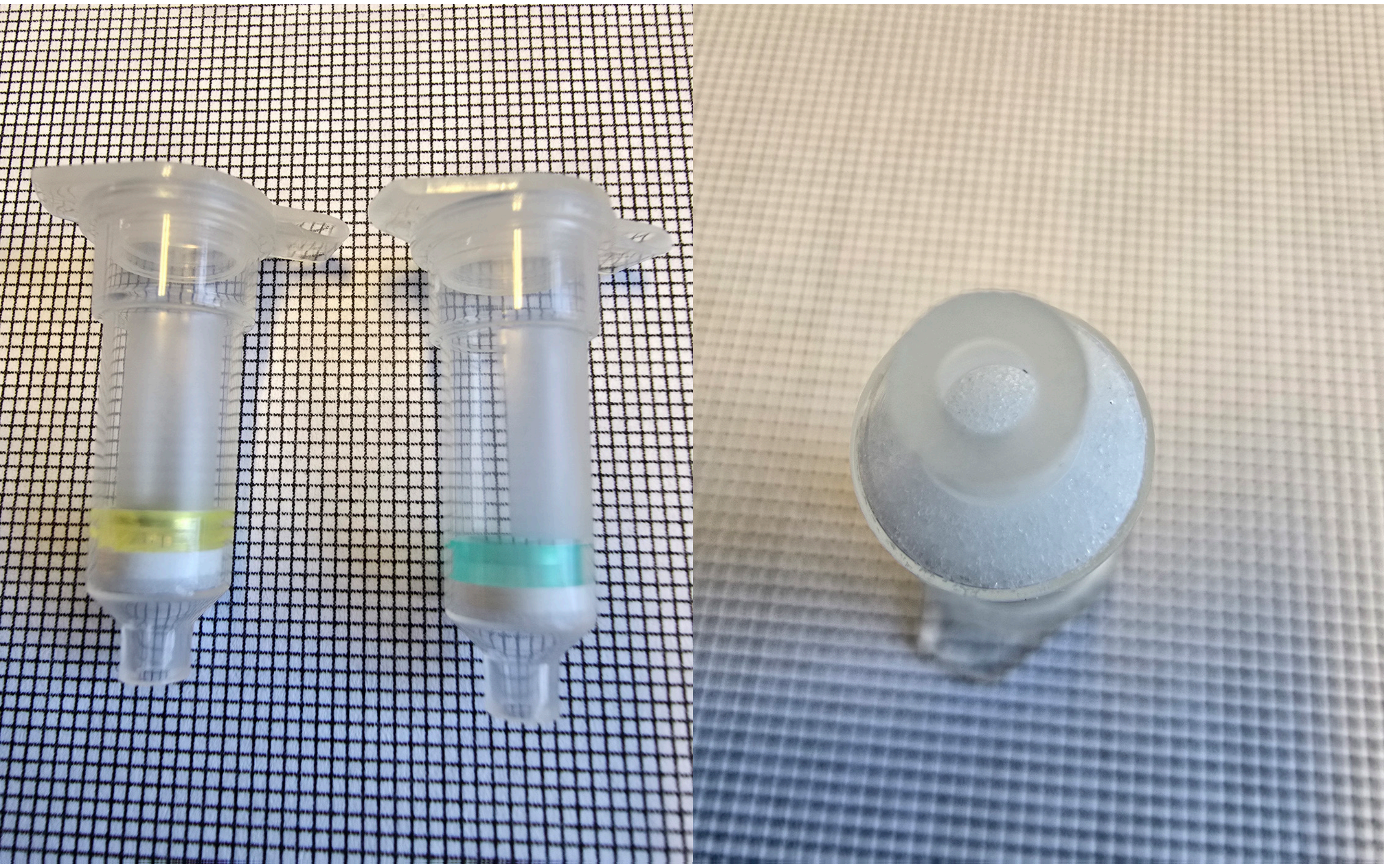

Quick-RNA MiniPrep columns
From left to right: Spin-Away Filters, Zymo-Spin IIICG Column, bottom of the Zymo-Spin IIICG Column

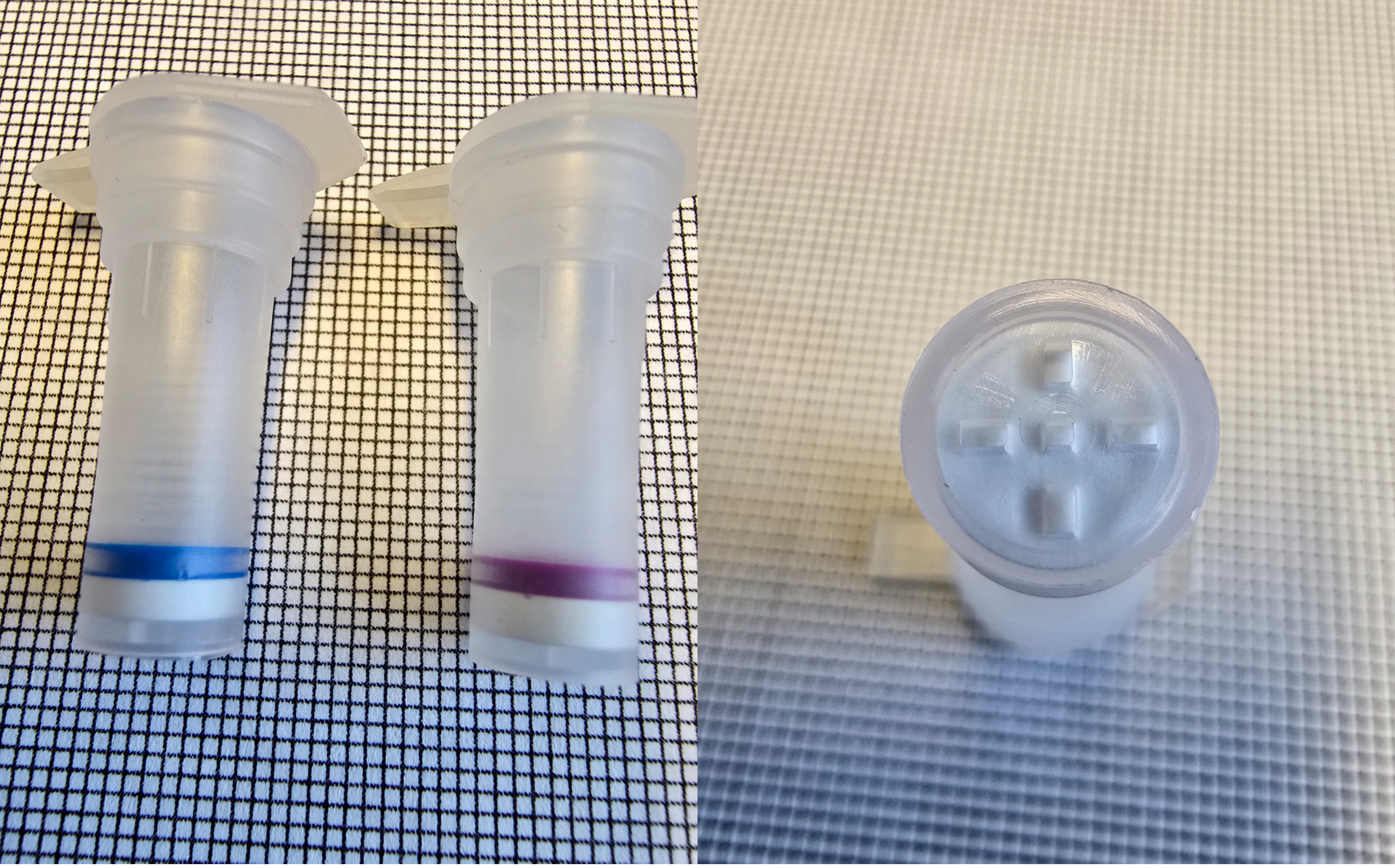

innuPREP RNA Mini Kit 2.0 columns
From left to right: Spin Filter D, Spin Filter R, bottom of the Spin Filter R
